# Efficacy of dynamic interpersonal therapy for major depressive disorder in China: results of a multicentered, three-arm, randomized, controlled trial

**DOI:** 10.1017/S0033291723000788

**Published:** 2023-11

**Authors:** Yuan Wang, Jiayu Yao, Diana Koszycki, Wenhui Jiang, Fang Fang, Minghong Wang, Jing Tao, Wenqing Zhao, Yilan Liu, Shanshan Su, Yihua Peng, Hongyan Wang, Lanlan Wang, Rui Gao, Junjie Gu, Jie Zhang, Yanle Bai, Yanru Wu, Yousong Su, Yating Zhao, Ziwei Zheng, Shuangyi Chen, Jianyin Qiu

**Affiliations:** 1 Shanghai Mental Health Center, Shanghai Jiao Tong University School of Medicine, Shanghai, China; 2 University of Ottawa and Institut du Savoir Montfort, Ottawa, Ontario, Canada; 3 Shanghai Hongkou District Mental Health Center, Shanghai, China; 4 Shanghai Changning District Mental Health Center, Shanghai, China; 5 Shanghai General Hospital Affiliated to Shanghai Jiao Tong University School of Medicine, Shanghai, China

**Keywords:** Dynamic interpersonal therapy, major depressive disorder, psychotherapy, randomized controlled trial

## Abstract

**Background:**

Dynamic interpersonal therapy (DIT) is a brief, structured psychodynamic psychotherapy with demonstrated efficacy in treating major depressive disorder (MDD). The aim of the study was to determine whether DIT is an acceptable and efficacious treatment for MDD patients in China.

**Method:**

Patients were randomized to 16-week treatments with either DIT plus antidepressant medication (DIT + ADM; *n* = 66), general supportive therapy plus antidepressant medication (GST + ADM; *n* = 75) or antidepressant medication alone (ADM; *n* = 70). The Hamilton Depression Rating Scale (HAMD) administered by blind raters was the primary efficacy measure. Assessments were completed during the acute 16-week treatment and up to 12-month posttreatment.

**Results:**

The group × time interaction was significant for the primary outcome HAMD (*F* = 2.900, df_1_ = 10, df_2_ = 774.72, *p* = 0.001) in the acute treatment phase. Pairwise comparisons showed a benefit of DIT + ADM over ADM at weeks 12 [least-squares (LS) mean difference = −3.161, *p* = 0.007] and 16 (LS mean difference = −3.237, *p* = 0.004). Because of the unexpected high attrition during the posttreatment follow-up phase, analyses of follow-up data were considered exploratory. Differences between DIT + ADM and ADM remained significant at the 1-, 6-, and 12-month follow-up (*p*s range from 0.001 to 0.027). DIT + ADM had no advantage over GST + ADM during the acute treatment phase. However, at the 12-month follow-up, patients who received DIT remained less depressed.

**Conclusions:**

Acute treatment with DIT or GST in combination with ADM was similarly efficacious in reducing depressive symptoms and yielded a better outcome than ADM alone. DIT may provide MDD patients with long-term benefits in symptom improvement but results must be viewed with caution.

## Introduction

Major depressive disorder (MDD) is a prevalent mental disorder, with a lifetime prevalence of 2–21% worldwide (Gutierrez-Rojas, Porras-Segovia, Dunne, Andrade-Gonzalez, & Cervilla, 2020). In China, the 12-month prevalence of MDD is 2.1% and the lifetime prevalence is 3.4% (Huang et al., 2019), with prevalence rates increasing by about 24% over the past three decades (Ren et al., 2020). In 2017, depression in China was the 10th leading cause of disability-adjusted life years (Ren et al., 2020; Zhou et al., 2019). The morbidity and mortality associated with MDD makes this disorder an important public health concern in China that deserves attention.

According to Chinese treatment guidelines pharmacotherapy and psychotherapy are recommended treatments for depressive disorders (Li & Ma, 2015). However, treatment utilization is very low in China, with less than 20% of patients receiving treatment for depression (Lu et al., 2021; Qi et al., 2019). Psychotherapy is underutilized in China and factors that are barriers to seeking and receiving psychological therapies include a shortage of treatment resources, high cost of treatment due to the relatively long treatment duration, and lack of standardized treatment implementation.

Psychodynamic psychotherapy is frequently used to treat MDD, with a growing body of empirical research supporting its efficacy (Connolly Gibbons et al., 2016; Driessen et al., 2015; Steinert, Munder, Rabung, Hoyer, & Leichsenring, 2017). However, few studies have evaluated the efficacy of psychodynamic psychotherapy for MDD in China. Traditional psychodynamic psychotherapy is usually delivered as a long-term and less-structured treatment, which may not be feasible in a country like China where mental health service resources are in short supply. It is therefore necessary to develop short-term and manualized psychodynamic psychotherapies to increase accessibility and feasibility as well as to reduce the costs. A suitable psychotherapy should be efficacious and easy to promote and deliver within the Chinese health care system. Dynamic interpersonal therapy (DIT) may be one potential intervention.

DIT has recently been developed as a 16-session manualized dynamic psychotherapy that is grounded in object relations, attachment, mentalization, and interpersonal theories (Lemma, Target, & Fonagy, 2011a, 2011b). In DIT, depression is conceptualized as a response to perceived threats to attachments (loss/separation) and impaired mentalization function. The most important content of DIT is the formulation and working through of a problematic, recurrent interpersonal-affective focus (IPAF). The IPAF describes the core interpersonal difficulties related to the patient's self and other representation in the attachment relationship. In other words, negative self-schema and other-schema are the background and play an important role in this process. The aims of DIT are to help the patient understand the connection between the onset and maintenance of depressive symptoms and the IPAF, and to facilitate the patient's capacity to reflect on their own state of mind so as to enhance their ability to manage interpersonal difficulties that underlie the symptoms of depression.

As one of the recommended psychotherapeutic approaches in the Improving Access to Psychological Therapies initiative in the UK (Chen, Ingenito, Kehn, Nehrig, & Abraham, 2019; Clark et al., 2018), DIT has been shown to be effective for patients with MDD in some studies (Chen, Nehrig, Wash, & Wang, 2020; Fonagy et al., 2020; Lemma et al., 2011b; Lemma, Target, & Fonagy, 2013). However, its efficacy and acceptability in depressed patients living in China has not been established. The objective of the current trial was to evaluate the efficacy and acceptability of DIT in treating MDD in China. We hypothesized that DIT plus antidepressant medication (ADM) would be superior to either ADM alone or general supportive therapy (GST) plus ADM in improving depression following acute treatment and throughout a 12-month posttreatment follow-up phase.

## Method

### Study design

The current study is a parallel group, multicentered randomized controlled trial (RCT) comparing DIT in combination with ADM (DIT + ADM), GST in combination with ADM (GST + ADM), and antidepressant medication alone (ADM). The study protocol was published previously (Wang et al., 2020). The trial was registered with the Chinese Clinical Trial Registry (ChiCTR1800016970) and the study is reported according to the CONSORT statement (Boutron et al., 2017; Moher et al., 2010).

### Participants

Outpatients were recruited from four hospitals in Shanghai, China [Shanghai Mental Health Center (SMHC; coordinating center), Shanghai General Hospital, Shanghai Hongkou District Mental Health Center, and Shanghai Changning District Mental Health Center]. In the original protocol (Wang et al., 2020) we planned to recruit participants from hospitals in Shanghai and three additional cities in China. However, the COVID-19 pandemic began shortly after the study was initiated and recruitment from centers outside of Shanghai was no longer possible. Therefore, we added two hospitals in Shanghai as new sites. The study was approved by the research ethics committee at each site. Written informed consent was obtained from participants before the screening assessment. Participants were recruited from April 2019 to December 2021.

**Fig. 1.**
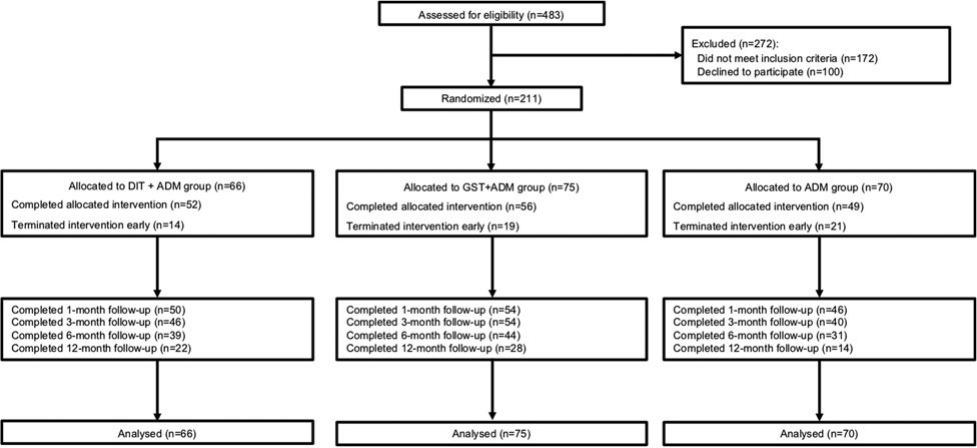
CONSORT diagram.

Patients were recruited via advertisement or psychiatrist referral. New patients were pre-screened individually for 15–20 min by a research assistant. Those who were considered potentially eligible were invited to attend the screen visit. In total, we assessed 483 patients for eligibility. Of these, 100 refused to participate and 172 were excluded because they did not meet the study inclusion criteria. The flowchart of participants during the trial is shown in Fig. 1.

Patients were included in the study if they (1) met DSM-5 criteria for MDD based on the Mini International Neuropsychiatric Interview (Sheehan et al., 1998), (2) had a score ⩾18 on the 17-item Hamilton Depression Rating Scale (HAMD) (Hamilton, 1960), (3) were between 18 and 65 years of age, (4) had at least primary school education (i.e. ⩾6 years of education), (5) were drug-free (i.e. had never taken any antidepressants or had discontinued antidepressants for at least 8 weeks prior to the screening visit), (6) did not receive any psychotherapy in the last 6 months, (7) were not treated by other approaches for depression, such as transcranial magnetic stimulation and electroconvulsive therapy, and (8) provided written informed consent.

Exclusion criteria included (1) a severe concurrent medical condition, (2) significant visual or auditory deficits, (3) a lifetime history of psychosis or bipolar disorder, (4) a history of substance use disorders in the last 24 months, (5) a history of psychotic features of affective disorder, (6) high suicide risk (MINI suicidality item score ⩾10), (7) severe personality disorders (e.g. cluster A and antisocial personality disorder) as determined by routine clinical interview by a psychiatrist, and (8) intellectual disability. Patients with other psychiatric comorbidities were included so long as these conditions were not more prominent than the MDD.

### Randomization and blinding

Eligible participants were randomized to one of the three treatments on a 1:1:1 basis via remote access to a central randomization procedure hosted by the Clinical Trial Center of SMHC. Study site was used as a stratified factor for stratified block randomization, and each site competed to enroll patients. Allocation of patients was performed by a research assistant who was not involved in recruitment and study assessments. The primary outcome was the HAMD administered by independent assessors who were blind to treatment allocation. Patients were asked not to divulge their assigned treatment during the evaluations. The principal researchers and statisticians were also blind to allocation of treatment.

### Treatment

Patients in all three treatment arms were prescribed selective serotonin reuptake inhibitors or serotonin norepinephrine reuptake inhibitors approved by the Chinese Food and Drug Administration for the treatment of MDD. These medications include paroxetine, sertraline, fluoxetine, fluvoxamine, citalopram, escitalopram, venlafaxine, and duloxetine. The choice of ADM was based on clinician recommendation and the dose could be titrated according to usual practice. The blind assessors documented medication use during the scheduled assessments. Patients were reminded to take their ADM as prescribed and were asked to record their daily use of medication as well as any other concomitant treatments.

DIT was based on the Chinese translation of the DIT manual developed by Lemma and colleagues (Lemma et al., 2011a). Patients received 16 weekly sessions with each session lasting 45 min. The treatment consists of three phases with specific tasks associated with each phase. The task of the initial phase of treatment (sessions 1–4) is to identify one dominant and recurring IPAF, which is the patient's unconscious interpersonal pattern that is presumed to underlie the patient's current depressive symptoms. The task of the middle phase (sessions 5–12) is to help the patient work through the IPAF. The therapist prioritizes the discussion of the patient's current relationships that activate the IPAF, using the therapeutic relationship as a live example of the IPAF in action, and helps the patient understand mental states as they relate to the week's events and to the identified IPAF. The final phase (sessions 13–16) focuses on working with the ending of therapy including exploring the patient's feelings about separation, discussing treatment gains, and anticipating future difficulties and vulnerabilities.

GST was also delivered as 16 weekly sessions with each session lasting 45 min. GST is an unstructured and non-directive psychotherapy that was used as an active control to rule out the benefits of common factors in psychological treatment. GST is patient-driven and not theoretically based, and involves the flexible use of basic psychotherapeutic techniques such as empathy, listening, noting and reflecting, eliciting affect, providing reassurance, and focusing on the patient's strengths. The goal of treatment is to strengthen coping resourcefulness and use of adaptive defense mechanisms to manage the demands of the patient's external and internal world. GST did not contain any key elements that are similar to DIT. It was carried out following the unpublished manual developed by our own research team (available from the senior author on request).

Among the randomized participants, 84.4% received treatments at the SMHC and 15.6% received treatment at the other sites.

### Therapists and treatment fidelity

Therapists were licensed psychotherapists with at least 3 years of psychotherapy experience. Therapists were trained in either DIT or GST before they could begin to treat study participants. Training in DIT and GST were conducted separately by experts in these approaches. DIT therapists attended a 5-day workshop that included 3 days of theoretical courses and 2 days of practical exercises. GST therapists attended a 3-day workshop that provided an overview of GST, a review of the GST treatment manual, and practical exercises. After training, only therapists who showed competency in either DIT or GST served as study therapists. Of these, 14 DIT therapists saw an average of 4.7 patients and 18 GST therapists saw an average of 4.2 patients. The study therapist attended a 3-h meeting every 3 months to review therapy-related knowledge. The therapists in each treatment group were equivalent with respect to number of years of psychotherapy experience (*t* = 0.190, *p* = 0.850) and professional training (Fisher's exact test *p* = 0.443).

Each therapist provided only one type of treatment and received 90-min group supervision every 2 weeks. Experienced therapists conducted the supervision to ensure the accuracy and consistency of treatment implementation. All of the DIT and GST sessions were audiotaped. For each patient, three sessions representing the beginning, middle, and end phase of therapy were reviewed by independent senior psychotherapists to establish treatment adherence and fidelity. We used the DIT session rating form in the clinician guide of DIT to assess the adherence (Lemma et al., 2011a). GST treatments were carried out according to the GST manual strictly, and the use of techniques related to DIT and any other specific psychotherapy was proscribed.

### Outcome

The primary outcome was the 17-item HAMD. Secondary outcomes included response, remission, and relapse rates based on the scores of HAMD-17, the Patient Health Questionnaire-9 (PHQ-9) (Spitzer, Kroenke, & Williams, 1999), Hamilton Anxiety Rating Scale (HAMA) (Hamilton, 1959), and Generalized Anxiety Disorder 7-item (GAD-7) (Spitzer, Kroenke, Williams, & Lowe, 2006). Response was defined as a 50% or greater reduction from baseline in total HAMD score, remission was defined as a HAMD score ⩽7, and relapse was defined as a HAMD score ⩾14 in those patients whose HAMD scores were less than 14 at posttreatment (Rush et al., 2006). To reduce measurement bias, the HAMD and the HAMA were administered centrally at SMHC by trained researchers. In the original protocol (Wang et al., 2020) we planned to conduct the assessments in person. However, due to COVID-19, the assessments had to be conducted by telephone or video call. Acute efficacy was measured at baseline and at weeks 2, 4, 8, 12, and 16. The PHQ-9 and GAD-7 were completed by patients every week during the acute treatment phase. Patients were assessed 1-, 3-, 6-, and 12-month posttreatment to examine maintenance of treatment gains.

### Sample size calculation

The power analysis was conducted by ‘G*Power’. A previous meta-analysis demonstrated that the effect sizes of short-term dynamic therapy were 0.71 and 0.34 (Anderson & Lambert, 1995). Additionally, considering the results of two other comparable studies (Fonagy et al., 2020; Jakobsen et al., 2017), the effect size assumed in our study was 0.50 with 80% power and 5% type I error rate. A sample size of 64 patients per group was considered sufficient to test the hypothesis that DIT + ADM would be superior to GST + ADM and ADM. Assuming a dropout rate of about 20%, our recruitment target was 80 patients in each group.

We planned to complete recruitment by April 2021. However, recruitment was negatively affected by the COVID-19 pandemic and the recruitment period was extended to December 2021. To avoid the discontinuity caused by excessive extension, which may affect the overall research progress, our final recruitment number in each group was close to but not over 80. No interim data analysis was performed that informed our decision to stop recruitment.

### Statistical analyses

One-way analysis of variance (ANOVA) and χ^2^ test were performed to examine group differences in baseline demographic and clinical characteristics, as well as the average dose of ADM during the 16-week treatment. Repeated measures of the primary and secondary continuous outcomes from baseline to session 16 were analyzed using linear-mixed models. Analysis was based on the intention-to-treat principle (ITT) with all randomized patients included in the analysis. The mixed model methodology, as opposed to conventional repeated-measures ANOVA, allows all available data on each patient to be used without having to use an imputation procedure. Treatment group, time, and interaction between treatment group and time were included as fixed effects. Age was included as a covariate in the model since it was the only demographic variable that differed between groups. Models also included the random effect of patients. We tested the model with the random effect of sites. However, site explained almost no variance in the model and was therefore not included in the final model. Restricted maximum likelihood was used to estimate the mixed models. In order to evaluate the significance of fixed effects in mixed models, Kenward–Roger approach was used to compute the degrees of freedom (Luke, 2017). The main interest in the linear-mixed model was whether improvement in outcome varied as a function of treatment. This would be reflected by a time-by-treatment interaction. Significant effects were followed with Bonferroni corrected pairwise comparisons. To assess maintenance of treatment gains the above analysis was repeated with the inclusion of baseline, 16-week as well as 1-, 3-, 6-, and 12-month posttreatment scores.

For categorical data, analyses of response, remission, and relapse rates were conducted for both the per protocol (PP) and ITT sample to ensure robustness of the results. For the PP sample, χ^2^ test was conducted to compare the response, remission, and relapse rates among the three groups. Considering the non-significant results on Little's missing completely at random test of the primary outcome HAMD score (χ^2^ = 94.710, *p* = 0.631), we assumed that the data were missed at random. Analyses for the ITT sample were used as sensitivity analyses. For the ITT sample, multiple imputation was used to handle missing data. Demographic variables (age, gender, marriage, and educational level), group, HAMD-17, and PHQ-9 at each time point were selected as predictors. Twenty imputed data sets were generated based on predictive mean matching. Logistic regression was performed on each data set with response, remission, and relapse rates as dependent variables and group as independent variable. The results from each data set were combined through Rubin's rule to obtain pooled results (Kleinke, Reinecke, & Spiess, 2020). Significance level was set at 0.05.

Linear-mixed models were performed with the packages ‘lme4’, ‘lmerTest’, and ‘emmeans’ in R (version 4.0.2). Multiple imputation was performed with the package ‘mice’ in R (version 4.0.2). Other statistical analyses were conducted in SPSS (version 24.0).

## Results

### Sample description

The flowchart of participants is shown in Fig. 1. Two hundred and eleven patients with MDD (*M*_age_ = 28.04, s.d._age_ = 6.23, 73.9% female) were randomized to either DIT + ADM (*n* = 66), GST + ADM (*n* = 75), or ADM (*n* = 70). The severity of depression and anxiety symptoms at baseline did not differ among the groups (Table 1). The groups differed with respect to age (*F* = 3.25, *p* = 0.041), with patients in the ADM group being younger than those in the GST + ADM group (*p* = 0.035). The groups did not differ across gender, marital status, and education level (Table 1).

**Table 1.** Participant demographic and clinical characteristics at baseline

	DIT + ADM (*n* = 66)		GST + ADM (*n* = 75)		ADM (*n* = 70)		*F*	*p*
	*M*	s.d.	*M*	s.d.	*M*	s.d.		
Age, years	28.09	5.44	29.18	5.84	26.67	7.07	3.25	0.041
HAMD total score	21.50	3.26	21.24	3.27	20.81	2.93	0.82	0.441
HAMA total score	16.03	3.18	16.32	3.52	16.24	3.61	0.13	0.877
PHQ total score	16.93	5.31	16.11	4.77	15.63	5.19	0.86	0.427
GAD total score	10.97	5.54	11.71	4.96	10.32	4.92	0.94	0.395
	*N*	%	*N*	%	*N*	%	χ^2^	*p*
Gender								
Male	15	22.7	17	22.7	23	32.9	2.07	0.286
Female	51	77.3	58	77.3	47	67.1		
Education								
Primary school	0	0	1	1.3	1	1.4	13.17	0.106
Secondary school	0	0	0	0	3	4.3		
High school	2	3.0	10	13.3	6	8.6		
Undergraduate	47	71.2	46	61.3	48	68.6		
Graduate	17	25.8	18	24.0	12	17.1		
Marriage								
Unmarried	46	69.7	44	58.7	52	74.3	7.82	0.252
Married	18	27.3	23	30.7	16	22.9		
Divorcee	2	3.0	7	9.3	2	2.9		
Separation	0	0	1	1.3	0	0		

DIT + ADM, DIT in combination with antidepressant medication group; GST + ADM, general supportive psychotherapy in combination with antidepressant medication group; ADM, antidepressant medication alone group; *M*, mean; s.d., standard deviation.

Fifty-four patients (25.6%) dropped out of acute treatment, and 157 (74.4%) completed the 16 weeks of treatment (DIT + ADM group: *n* = 52; GST + ADM group: *n* = 56; and ADM group: *n* = 49). The attrition rate was comparable across the three groups 21.2% (*n* = 14) for DIT + ADM, 25.3% (*n* = 19) for GST + ADM, and 30% (*n* = 21) for ADM (χ^2^ = 1.38, *p* = 0.501).

The average equivalent dose (i.e. dose that would be equivalent to 20 mg fluoxetine) (Furukawa et al., 2019; Perahia et al., 2008) of ADM among the three treatment groups during the 16-week treatment did not differ significantly (DIT + ADM group: 27.10 ± 8.50 mg; GST + ADM group: 29.61 ± 10.89 mg; ADM group: 30.67 ± 18.54 mg; *F* = 1.15, *p* = 0.319). At the end of the 16-week treatment, 68.3% patients in the DIT + ADM group, 69.4% patients in the GST + ADM group, and 56.7% patients in the ADM group were still taking antidepressants, indicating that adherence to medication was comparable among the three groups (χ^2^ = 2.93, *p* = 0.235).

### Acute treatment effects

**Fig. 2.**
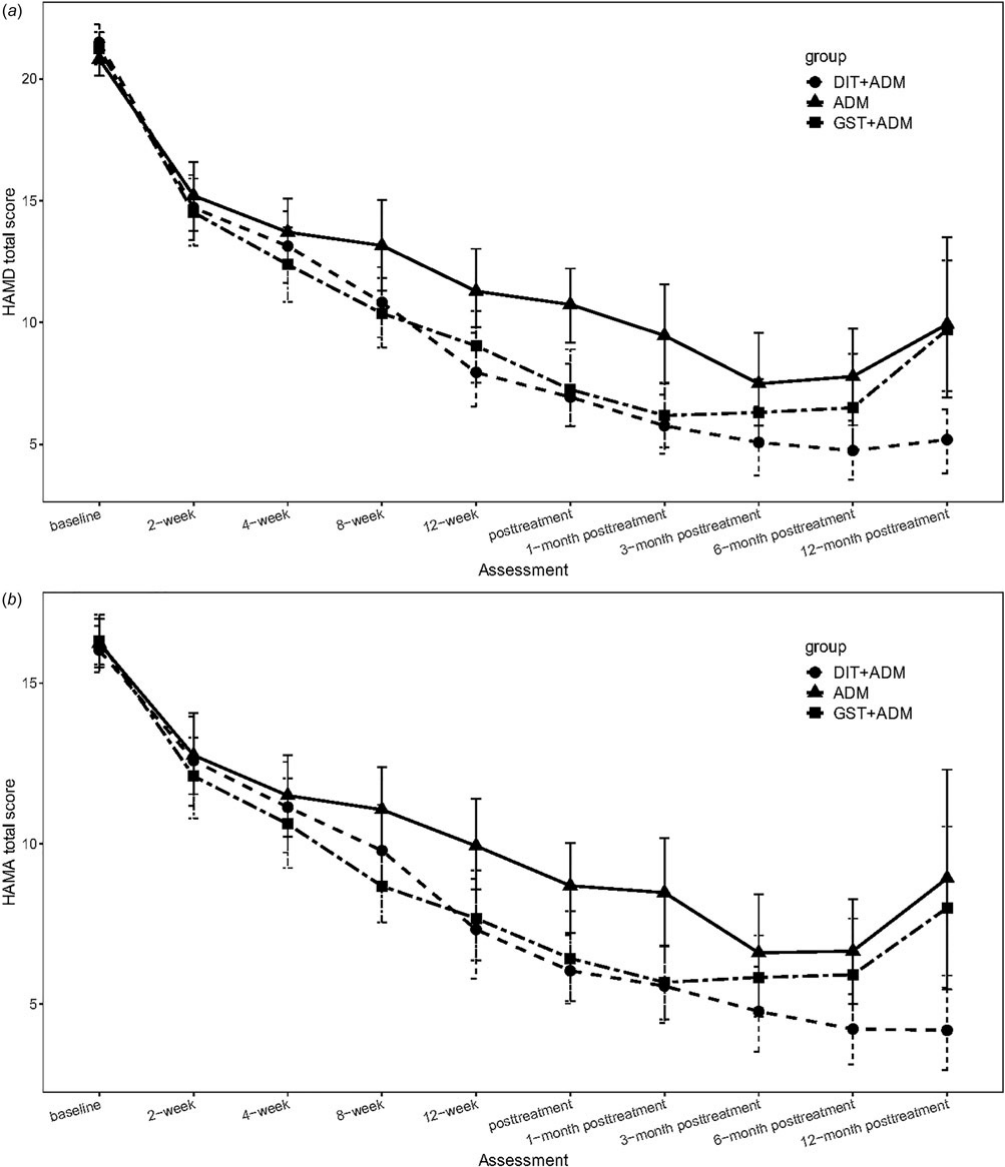
Comparison of HAMD and HAMA scores among the three treatment groups. DIT + ADM, DIT in combination with antidepressant medication group; GST + ADM, general supportive psychotherapy in combination with antidepressant medication group; ADM, antidepressant medication alone group.

The group × time interaction was significant (*F* = 2.90, df_1_ = 10, df_2_ = 774.72, *p* = 0.001) for the primary outcome HAMD. Pairwise comparisons showed a benefit of DIT + ADM over ADM at weeks 12 [least-squares (LS) mean difference = −3.16, *p* = 0.007] and 16 (LS mean difference = −3.24, *p* = 0.004). There was also a benefit of GST + ADM over ADM on HAMD scores at weeks 8 (LS mean difference = −2.70, *p* = 0.014) and 16 (LS mean difference = −3.37, *p* = 0.002). DIT + ADM had no advantage over GST + ADM in reducing HAMD scores (Table 2, Fig. 2).

**Table 2.** Comparison of scores of HAMD, PHQ-9, HAMA, and GAD-7 among the three treatment groups (ITT analysis) during acute treatment and 12-month follow-up posttreatment

	DIT + ADM	GST + ADM	ADM	Group × time			(DIT + ADM) *v.* ADM	(GST + ADM) *v.* ADM	(DIT + ADM) *v.* (GST + ADM)
	*M* (s.d.)	*M* (s.d.)	*M* (s.d.)	*F*	df_1, 2_	*p*	LS mean difference (*p*)	LS mean difference (*p*)	LS mean difference (*p*)
HAMD									
Acute treatment phase				2.90	10, 774.72	0.001**			
2 weeks	14.71 (5.38)	14.52 (5.39)	15.21 (5.23)				−0.59 (1.000)	−0.64 (1.000)	0.05 (1.000)
4 weeks	13.71 (5.28)	12.38 (6.00)	13.71 (5.22)				−0.40 (1.000)	−0.85 (1.000)	0.44 (1.000)
8 weeks	10.82 (5.16)	10.38 (5.24)	13.16 (6.30)				−2.24 (0.058)	−2.70 (0.014)*	0.46 (1.000)
12 weeks	7.95 (5.21)	9.04 (5.57)	11.29 (5.31)				−3.16 (0.007)**	−2.12 (0.098)	−1.04 (0.880)
16 weeks	6.94 (4.59)	7.25 (5.51)	10.74 (5.14)				−3.24 (0.004)**	−3.37 (0.002)**	0.14 (1.000)
Posttreatment follow-up phase				3.74	10, 593.67	<0.001**			
1-month posttreatment	5.76 (4.09)	6.18 (4.43)	9.46 (6.50)				−3.69 (0.001)**	−2.94 (0.012)*	−0.75 (1.000)
3-month posttreatment	5.08 (4.60)	6.31 (5.34)	7.49 (5.83)				−2.41 (0.070)	−1.05 (0.907)	−1.36 (0.503)
6-month posttreatment	4.74 (3.61)	6.50 (5.93)	7.79 (5.52)				−3.64 (0.006)**	−1.42 (0.645)	−2.22 (0.139)
12-month posttreatment	5.19 (3.09)	9.70 (7.50)	9.93 (6.51)				−3.97 (0.027)*	0.36 (1.000)	−4.33 (0.003)**
PHQ-9									
Acute treatment phase				3.85	32, 2093.58	<0.001**			
2 weeks	12.91 (6.15)	11.16 (4.90)	14.00(5.69)				−1.156(0.940)	−2.71 (0.050)	1.56 (0.351)
4 weeks	9.92 (5.36)	9.47 (5.92)	11.31 (5.75)				−1.94 (0.271)	−2.47 (0.085)	0.53 (1.000)
8 weeks	7.36 (5.35)	8.41 (5.75)	11.80 (6.97)				−3.89 (0.003)**	−3.123 (0.019)*	−0.76 (1.000)
12 weeks	6.35 (5.09)	7.31 (5.84)	10.84 (6.54)				−4.22 (0.001)**	−3.96 (0.003)**	−0.26 (1.000)
16 weeks	5.46 (4.35)	6.93 (5.42)	10.97 (6.62)				−5.39 (<0.001)**	−4.80 (<0.001)**	−0.58 (1.000)
Posttreatment follow-up phase				4.00	10, 439.04	<0.001**			
1-month posttreatment	7.42 (5.94)	5.97 (3.59)	11.44(6.20)				−3.29 (0.035)*	−3.84 (0.009)**	0.55 (1.000)
3-month posttreatment	6.43 (4.58)	7.61 (4.98)	9.45 (5.76)				−3.32 (0.048)*	−1.60 (0.684)	−1.71 (0.476)
6-month posttreatment	6.65 (5.631)	8.58 (5.57)	8.28 (6.64)				−2.66 (0.256)	0.19 (1.000)	−2.85 (0.134)
12-month posttreatment	6.42 (3.75)	8.38 (5.88)	9.40 (6.29)				−2.96 (0.443)	−0.23 (1.000)	−2.72 (0.300)
HAMA									
Acute treatment phase				1.72	10, 771.28	0.072			
2 weeks	12.59 (5.54)	12.11 (4.81)	12.75 (4.75)				–	–	–
4 weeks	11.14 (5.11)	10.61 (5.40)	11.50 (4.69)				–	–	–
8 weeks	9.78 (4.90)	8.68 (4.26)	11.06 (4.80)				–	–	–
12 weeks	7.33 (5.25)	7.67 (4.91)	9.93 (4.82)				–	–	–
16 weeks	6.04 (3.91)	6.43 (5.01)	8.69 (4.90)				–	–	–
Posttreatment follow-up phase				2.14	10, 589.08	0.020*			
1-month posttreatment	5.57 (4.25)	5.68 (4.18)	8.48 (5.58)				−2.79 (0.009)**	−2.23 (0.053)	−0.56 (1.000)
3-month posttreatment	4.78 (4.47)	5.84 (4.71)	6.60(5.26)				−1.65 (0.286)	−0.37 (1.000)	−1.27 (0.493)
6-month posttreatment	4.23 (3.35)	5.92 (5.38)	6.66 (4.73)				−2.38 (0.082)	−0.02 (1.000)	−2.37 (0.064)
12-month posttreatment	4.19 (3.04)	8.00 (6.25)	8.92 (6.37)				−2.87 (0.130)	0.98 (1.000)	−3.85 (0.004)**
GAD-7									
Acute treatment phase				3.11	32, 2096.17	<0.001**			
2 weeks	8.23 (5.61)	8.02 (4.75)	9.59 (5.48)				−1.62 (0.349)	−1.76 (0.250)	0.14 (1.000)
4 weeks	6.89 (4.88)	6.07 (4.56)	7.38 (5.28)				−1.08 (0.890)	−2.21 (0.092)	1.13 (0.650)
8 weeks	5.12 (4.30)	8.03 (6.06)	5.87 (4.21)				−2.13 (0.128)	−1.84 (0.226)	−0.29 (1.000)
12 weeks	4.08 (4.12)	5.65 (4.21)	8.12 (6.54)				−3.12 (0.012)*	−2.21 (0.122)	−0.91 (1.000)
16 weeks	3.02 (2.79)	4.80 (3.99)	7.28 (5.53)				−4.18 (<0.001)**	−3.44 (0.004)**	−0.75 (1.000)
Posttreatment follow-up phase				2.52	10, 443.24	0.006**			
1-month posttreatment	4.52 (4.99)	5.39 (4.28)	8.81 (5.48)				−3.63 (0.008)**	−2.40 (0.120)	−1.23 (0.812)
3-month posttreatment	4.10 (3.48)	6.14 (5.11)	6.77 (5.06)				−2.55 (0.134)	−0.83 (1.000)	−1.72 (0.370)
6-month posttreatment	4.80 (3.69)	6.96 (5.35)	5.39 (6.20)				−0.84 (1.000)	1.13 (1.000)	−1.98 (0.396)
12-month posttreatment	4.33 (3.31)	6.79 (5.53)	7.80 (5.75)				−3.06 (0.316)	−0.47 (1.000)	−2.59 (0.272)

*M* and s.d. are the observed mean and standard deviation of the data. The *F* and *p* values for fixed effects are calculated with Kenward–Roger approach to estimate degrees of freedom. LS mean difference is the estimate of the differences between each comparison of least-squares means. DIT + ADM, DIT in combination with antidepressant medication group; GST + ADM, general supportive psychotherapy in combination with antidepressant medication group; ADM, antidepressant alone group.

*p < 0.05; **p < 0.01.

Analysis of secondary outcomes revealed a significant time × group interaction for the PHQ-9 (*F* = 3.85, df_1_ = 32, df_2_ = 2093.58, *p* < 0.001) and GAD-7 (*F* = 3.11, df_1_ = 32, df_2_ = 2096.17, *p* < 0.001). There was no significant time × group interaction for the HAMA (*F* = 1.72, df_1_ = 10, df_2_ = 771.28, *p* = 0.072). Pairwise comparisons revealed an advantage of DIT + ADM and GST + ADM over ADM in reducing PHQ-9 scores at week 8 (DIT + ADM *v.* ADM *p* = 0.003, GST + ADM *v.* ADM *p* = 0.019), week 12 (DIT + ADM *v.* ADM *p* = 0.001, GST + ADM *v.* ADM *p* = 0.003), and week 16 (DIT + ADM *v.* ADM *p* < 0.001, GST + ADM *v.* ADM *p* < 0.001), and in reducing GAD-7 scores at week 12 (DIT + ADM *v.* ADM *p* < 0.012), and week 16 (DIT + ADM *v.* ADM *p* < 0.001, GST + ADM *v.* ADM *p* = 0.004). DIT + ADM had no advantage over GST + ADM in decreasing PHQ-9 or GAD-7 scores (Table 2, Fig. 2).

The response rate differed among the three groups at posttreatment (χ^2^ = 14.28, *p* < 0.001) (Table 3). Response rates were higher in the DIT + ADM (*p* = 0.006) and GST + ADM (*p* = 0.007) group than the ADM group. Sensitivity analyses after imputation showed the same results.

**Table 3.** Comparison of response and remission rates among the three treatment groups

	DIT + ADM		GST + ADM		ADM				(DIT + ADM) *v*. ADM	(GST + ADM) *v*. ADM	(DIT + ADM) *v*. (GST + ADM)
	*N*	%	*N*	%	*N*	%	χ^2^	*p*	*p*	*p*	*p*
Posttreatment (16-weeks)											
Response							14.28	<0.001**	0.006**	0.007**	1.000
HAMD reduce rate >50%	37	78.72	37	77.08	19	45.24					
HAMD reduce rate ⩽50%	10	21.28	11	22.92	23	54.76					
Remission							7.83	0.020*	0.056	0.060	1.000
HAMD total score ⩽7	27	57.45	27	56.25	13	30.95					
HAMD total score >7	20	42.55	21	43.75	29	69.05					
1-month posttreatment											
Response							12.85	0.002**	0.003**	0.098	0.760
HAMD reduce rate >50%	41	89.13	35	79.55	22	56.41					
HAMD reduce rate ⩽50%	5	10.87	9	20.45	17	43.59					
Remission							5.56	0.062	0.093	0.241	1.000
HAMD total score ⩽7	31	67.39	28	63.64	17	43.59					
HAMD total score >7	15	32.61	16	36.36	22	56.41					
3-month posttreatment											
Response							3.95	0.139	0.162	1.000	0.824
HAMD reduce rate >50%	35	87.50	38	77.55	24	68.57					
HAMD reduce rate ⩽50%	5	12.50	11	22.45	11	31.43					
Remission							2.95	0.229	0.674	0.479	1.000
HAMD total score ⩽7	29	72.50	36	73.47	20	57.14					
HAMD total score >7	11	27.50	13	26.53	15	42.86					
6-month posttreatment											
Response							14.99	0.001**	<0.001**	0.334	0.048*
HAMD reduce rate >50%	30	96.77	27	75.00	15	53.57					
HAMD reduce rate ⩽50%	1	3.23	9	25.00	13	46.43					
Remission							6.14	0.046*	0.079	0.940	0.529
HAMD total score ⩽7	25	80.65	23	63.89	14	50.00					
HAMD total score >7	6	19.35	13	36.11	14	50.00					
12-month posttreatment											
Response							7.00	0.030*	0.269	1.000	0.033*
HAMD reduce rate >50%	19	90.48	15	55.56	9	64.29					
HAMD reduce rate ⩽50%	2	9.52	12	44.44	5	35.71					
Remission							6.74	0.034*	0.225	1.000	0.060
HAMD total score ⩽7	16	76.19	11	40.74	6	42.86					
HAMD total score >7	5	23.81	16	59.26	8	57.14					

Number (*n*), χ^2^, and *p* values are calculated with the data before multiple imputation.

DIT + ADM, DIT in combination with antidepressant medication group; GST + ADM, general supportive psychotherapy in combination with antidepressant medication group; ADM, antidepressant medication alone group.

*p < 0.05; **p < 0.01.

The remission rate was differed among the three groups at posttreatment (χ^2^ = 7.83, *p* = 0.020) (Table 3). There was no significant difference in pairwise comparisons. Sensitivity analyses after imputation showed the same results.

### Maintenance of treatment gains

Participants were followed for 12 months after the 16 weeks of acute treatment to assess maintenance of treatment gains. However, the attrition rate was relatively high. At 6-month posttreatment, the attrition rate was 40.9% (*n* = 27) for DIT + ADM, 41.3% (*n* = 31) for GST + ADM, and 55.7% (*n* = 39) for ADM (χ^2^ = 4.01, *p* = 0.135). At 12-month posttreatment, the attrition rate was 66.7% (*n* = 44) for DIT + ADM, 62.7% (*n* = 47) for GST + ADM, and 80% (*n* = 56) for ADM (χ^2^ = 5.56, *p* = 0.062). Because of the higher than planned attrition rate during the follow-up phase of the study, analysis of maintenance data was considered exploratory. At 12-month posttreatment, 48.5% patients in the DIT + ADM group, 58.7% patients in the GST + ADM group, and 41.4% patients in the ADM group were still taking antidepressants (χ^2^ = 4.69, *p* = 0.321). Few patients received any other type of psychotherapy.

Linear-mixed models of posttreatment follow-up data on HAMD scores revealed a significant group × time interaction (*F* = 3.74, df_1_ = 10, df_2_ = 593.67, *p* < 0.001). Pairwise comparisons showed that with the exception of the 3-month follow-up (LS mean difference = −2.41, *p* = 0.070), DIT + ADM continued to have an advantage over ADM at 1-month (LS mean difference = −3.69, *p* = 0.001), 6-month (LS mean difference = −3.64, *p* = 0.006), and 12-month (LS mean difference = −3.97, *p* = 0.027) posttreatment. DIT + ADM also had an advantage over GST + ADM at the 12-month follow-up (LS mean difference = −4.33, *p* = 0.003). GST + ADM did not continue to have any advantage over ADM during the follow-up period except at the 1-month follow-up (LS mean difference = −2.94, *p* = 0.012) (Table 2, Fig. 2)

Analysis of secondary data revealed a significant group × time interaction for the PHQ-9 (*F* = 4.00, df_1_ = 10, df_2_ = 439.04, *p* < 0.001), HAMA (*F* = 2.14, df_1_ = 10, df_2_ = 589.08, *p* = 0.020), and GAD-7 (*F* = 2.52, df_1_ = 10, df_2_ = 443.24, *p* = 0.006). For the PHQ-9, DIT + ADM continued to have an advantage over ADM at 1-month (*p* = 0.035) and 3-month (*p* = 0.048) posttreatment, while the difference between GST + ADM and ADM was significant at 1-month posttreatment (*p* = 0.009). For the HAMA, the difference between DIT + ADM and ADM was significant at 1-month (*p* = 0.009) posttreatment. DIT + ADM had an advantage over GST + ADM in reducing HAMA scores at 12-month posttreatment (*p* = 0.004). For the GAD-7, DIT + ADM had an advantage over ADM at 1-month posttreatment (*p* = 0.008) (Table 2, Fig. 2).

Analysis of maintenance effects for response, remission, and relapse rates showed that rates of response among the three groups were significantly different at 1-month (χ^2^ = 12.85, *p* = 0.002), 6-month (χ^2^ = 14.99, *p* = 0.001), and 12-month posttreatment (χ^2^ = 7.00, *p* = 0.030). The response rates were higher in the DIT + ADM *v.* ADM group at 1-month (*p* = 0.003) and 6-month posttreatment (*p* < 0.001). Response rates were higher in the DIT + ADM *v.* GST + ADM group at 6-month (*p* = 0.048) and 12-month (*p* = 0.033) posttreatment. Sensitivity analyses after imputation showed the same results (Table 3).

The rates of remission among the three groups were significantly different at 6-month (χ^2^ = 6.14, *p* = 0.046) and 12-month (χ^2^ = 6.74, *p* = 0.034) posttreatment (Table 3). Sensitivity analyses after imputation showed almost the same results except that the remission rates in the DIT + ADM condition were significantly higher than the ADM condition at 6-month (*p* = 0.023) posttreatment. GST + ADM showed no difference in remission rates relative to ADM in the follow-up phase (data are shown in the online Supplementary materials).

The rates of relapse did not differ among the groups based on the ITT and PP analyses (data are shown in the online Supplementary materials).

## Discussion

This is the first multicentered RCT in China to assess the efficacy of DIT for MDD. As expected, participants randomized to DIT + ADM were less depressed following acute treatment than those who received ADM alone. Remission was achieved in 57.45% of patients and 78.72% patients showed response to the treatment. We used an active control of GST + ADM in this study. GST + ADM also yielded improvement in depression that was superior to ADM, with remission and response rates comparable to that in DIT + ADM.

The results of our study are consistent with previous studies of DIT. The acceptability and effectiveness of DIT for depression was reported in an initial pilot study by Lemma and colleagues (Lemma et al., 2011b). Large pre-to-posttreatment effect sizes were found for measures of depression and anxiety, with 70% of the patients reporting symptoms below clinical levels (Lemma et al., 2013). In another study (Wright & Abrahams, 2015), 75% of patients treated with DIT showed significant reductions in self-report depression and anxiety. However, the significance in this study cannot be reliably attributed to DIT because it was an uncontrolled trial. DIT has also been found to be efficacious in reducing depression and anxiety in veterans (Chen et al., 2020). A recent RCT of DIT conducted by Fonagy and colleagues found that DIT was superior to low-intensity treatment, which was a self-guided manual-based treatment, and comparable to cognitive behavior therapy in treating depression (Fonagy et al., 2020).

In our study, both DIT and GST combined with ADM yielded improvement in depression that was superior to ADM alone. This result may be explained by the extra benefit of combining psychotherapy and medication. Indeed, the most recent meta-analysis exploring the effects of psychotherapy, pharmacotherapy, and their combination in the treatment of depression confirmed the superiority of combined treatment for major depression, chronic depression, and treatment-resistant depression (Cuijpers et al., 2020). Combined treatment might have better long-term effects (Cuijpers et al., 2020) although it is difficult to determine the underling mechanism of the clinical advantage of combined treatment. One possible explanation is that psychotherapy improves compliance with drug treatment and patients are more satisfied with combined treatment than medication alone (Pampallona, Bollini, Tibaldi, Kupelnick, & Munizza, 2004). Studies have shown that patients in long-term psychotherapy are less likely to drop out of treatment if psychotherapy is combined with medication (Cuijpers, Dekker, Hollon, & Andersson, 2009; Pampallona et al., 2004). Further studies should explore whether there are additional specific benefits of combining psychotherapy and medication other than its compliance-enhancing effect (Pampallona et al., 2004), or whether the benefits of psychotherapy and pharmacotherapy in combination are separate from each other.

In the current study, DIT + ADM was not superior to GST + ADM in reducing symptoms of depression in the acute treatment phase. There are few comparative trials of psychotherapies for depression and existing studies are underpowered and suffer from bias, making it difficult to determine the difference of effects among different types of psychotherapies (Cuijpers, 2016). A recent network meta-analysis of psychological therapies for depression concluded that non-directive supportive counseling was less effect than other treatments for depression, including cognitive behavior therapy, interpersonal therapy, psychodynamic therapy, problem-solving therapy, behavioral activation, life-review, and ‘third wave’ therapies, (Cuijpers et al., 2021). However, the inferiority of non-directive supportive counseling was no longer significant when studies with high risk of bias were excluded (Cuijpers et al., 2021). In another meta-analysis, non-directive supportive therapy was found to be effective for treating adult depression (Cuijpers et al., 2012). The contribution of extra-therapeutic factors, non-specific common factors, and specific therapy factors for improvement in depressive symptoms was 33.3, 49.6, and 17.1%, respectively (Cuijpers et al., 2012). Considering that half of the total effects in psychotherapy is due to non-specific common factors, it is not surprising that DIT and GST were both effective as an acute treatment for depression in this study.

Treatment gains were maintained at follow-up, with depression scores remaining lower for patients who received DIT + ADM *v.* ADM at 1-, 6-, and 12-month follow-up assessments. In contrast, the benefits of GST + ADM over ADM were less enduring during all but the 1-month follow-up time point. As well, at 12-months posttreatment, GST + ADM had lost its effects relative to DIT + ADM. A previous meta-analysis showed that the combination of short-term psychodynamic psychotherapy (STPP) and antidepressants was more effective than antidepressants with or without brief supportive psychotherapy in treating depression at both posttreatment and follow-up, which is partly consistent with our results from the current study (Driessen et al., 2020). STPP was found to be more effective during the follow-up phase than at posttreatment (Driessen et al., 2020). The trend of emerging advantages of STPP over time is consistent with our findings. One possible explanation for this better enduring effect is that patients receiving short-term psychodynamic therapies like DIT moderate their implicit memory and core schemas during the treatment. It can be regarded as an experience-learning process and the effects continue to work even after treatment ends. However, it is important to emphasize that conclusions about the possible enduring effect of DIT should be made with caution due to the considerable attrition during the follow-up phase of this study. Longitudinal data with low attrition are needed to determine whether DIT has a long-term benefit.

DIT is a psychotherapy mainly developed to improve symptoms of depression and anxiety. However, research findings have mainly focused on depression. Our study found that DIT + ADM reduced self-report levels of anxiety relative to ADM during the acute treatment phase, but benefits did not continue beyond the 1-month follow-up. Further research is needed to determine whether DIT is of potential benefit in the treatment of anxiety symptoms.

There were several strengths in our study. This is the first RCT study of psychodynamic psychotherapy conducted in China that examined the efficacy of DIT in the treatment of MDD. We used GST to control for the confounding effects of common elements of psychotherapy, making our research conclusions more accurate. We designed a relatively long follow-up period (12-month posttreatment) to explore the stability of acute treatment effects.

The study also had several limitations. First, because of the influence of COVID-19, the dropout rate was higher than expected, especially during the posttreatment follow-up phase. This introduced an important bias to the study and limited the reliability of posttreatment follow-up data and conclusions that can be drawn about enduring effects of DIT. Second, we focused on the evaluation of clinical symptoms and did not assess treatment acceptability and patients' social functioning or explore more psychological mechanisms. Third, we did not measure non-specific therapy effects, such as the therapeutic alliance and perception of treatment credibility, as well as the influence of therapist factors (Lewis, Locke, Heritage, & Seddon, 2022), which may have influenced the efficacy of different treatments. Fourth, we did not use questionnaires to assess personality and the impact of personality pathology on treatment response. Moreover, the study sample was limited to depressed people who lived in Shanghai and cities nearby. China is a country with unbalanced economic development and obvious cultural diversity, which affects the generalization of results to a certain extent. In the future, research on the efficacy and acceptability of DIT should be conducted in different regions of China. Finally, cost-effectiveness analyses were not performed in the study and this would be worthwhile to explore in future research with DIT.

## Conclusion

This is the first multicentered RCT of DIT for MDD conducted in China. Sixteen weekly sessions of DIT + ADM resulted in a greater improvement in depressive symptom than ADM alone, but similar improvement to GST + ADM. There was preliminary evidence that DIT may provide patients with MDD with long-term benefits in symptom improvement.

## Supporting information

Wang et al. supplementary material 1 Wang et al. supplementary material

Wang et al. supplementary material 2 Wang et al. supplementary material

## Supplementary material

The supplementary material for this article can be found at https://doi.org/10.1017/S0033291723000788.
