# Supplementary material for: Efficacy of dynamic interpersonal therapy for major depressive disorder in China: results of a multicentered, three-arm, randomized, controlled trial
Source: Psychol Med. 2023 Apr 5;53(15):7242–54. doi: 10.1017/S0033291723000788 (PMC10719685; doi:10.1017/S0033291723000788)
Supplement: Wang et al. supplementary material 1 — Wang et al. supplementary material [file S0033291723000788sup001.pdf]

Table 1 (Supplementary). Comparison of remission and response rates among three treatment groups (analyses after imputation)

		DIT+ADM		GST+ADM		ADM		(DIT+ADM) vs ADM	(GST+ADM) vs ADM	(DIT+ADM) vs (GST+ADM)
		N	%	N	%	N	%	etimate, std.error, *p*	etimate, std.error, *p*	etimate, std.error, *p*
Posttreatment (16-week)	Response							-1.25,0.48,0.012**	-1.06,0.46,0.026*	-0.18,0.48,0.703
	HAMD reduce rate >50%	37	78.72	37	77.08	19	45.24			
	HAMD reduce rate ⩽ 50%	10	21.28	11	22.92	23	54.76			
	Remission							-0.83,0.44,0.065	-0.86,0.45,0.063	0.03,0.39,0.944
	HAMD total score ⩽ 7	27	57.45	27	56.25	13	30.95			
	HAMD total score >7	20	42.55	21	43.75	29	69.05			
1-month posttreatment	Response							-1.41,0.51,0.007**	-0.68,0.42,0.110	-0.74,0.51,0.148
	HAMD reduce rate >50%	41	89.13	35	79.55	22	56.41			
	HAMD reduce rate ⩽ 50%	5	10.87	9	20.45	17	43.59			
	Remission							-0.75,0.42,0.079	-0.49,0.38,0.200	-0.26,0.40,0.519
	HAMD total score ⩽ 7	31	67.39	28	63.64	17	43.59			
	HAMD total score >7	15	32.61	16	36.36	22	56.41			
3-month posttreatment	Response							-0.76,0.58,0.198	-0.24,0.46,0.603	-0.52,0.58,0.374
	HAMD reduce rate >50%	35	87.50	38	77.55	24	68.57			
	HAMD reduce rate ⩽ 50%	5	12.50	11	22.45	11	31.43			
	Remission							-0.46,0.45,0.313	-0.42,0.44,0.351	-0.04,0.46,0.925
	HAMD total score ⩽ 7	29	72.50	36	73.47	20	57.14			
	HAMD total score >7	11	27.50	13	26.53	15	42.86			
6-month posttreatment	Response							-1.86,0.66,0.007**	-0.54,0.47,0.260	-1.32,0.65,0.046*
	HAMD reduce rate >50%	30	96.77	27	75.00	15	53.57			

	HAMD reduce rate ≤ 50%	1	3.23	9	25.00	13	46.43		
	Remission							-1.17,0.50,0.023*	-0.35,0.43,0.417	-0.82,0.49,0.095
	HAMD total score ≤ 7	25	80.65	23	63.89	14	50.00			
	HAMD total score >7	6	19.35	13	36.11	14	50.00			
12-month posttreatment	Response							-1.26,0.63,0.056	-0.14,0.63,0.828	-1.12,0.55,0.048*
	HAMD reduce rate >50%	19	90.48	15	55.56	9	64.29			
	HAMD reduce rate ≤ 50%	2	9.52	12	44.44	5	35.71			
	Remission							-1.06,0.65,0.117	-0.18,0.61,0.772	-0.88,0.51,0.091
	HAMD total score ≤ 7	16	76.19	11	40.74	6	42.86			
	HAMD total score >7	5	23.81	16	59.26	8	57.14			

DIT + ADM, DIT in combination with antidepressant medication group; GST + ADM, general supportive psychotherapy in combination with antidepressant medication group; ADM, antidepressant medication alone group.

*p<0.05; **p<0.01.
