# Supplementary material for: Efficacy of dynamic interpersonal therapy for major depressive disorder in China: results of a multicentered, three-arm, randomized, controlled trial
Source: Psychol Med. 2023 Apr 5;53(15):7242–54. doi: 10.1017/S0033291723000788 (PMC10719685; doi:10.1017/S0033291723000788)
Supplement: Wang et al. supplementary material 2 — Wang et al. supplementary material [file S0033291723000788sup002.pdf]

Table 2 (Supplementary). Comparison of relapse rates among three treatment groups

		analyses before imputation			analyses after imputation		
		(DIT+ADM) vs ADM	(GST+ADM) vs ADM	(DIT+ADM) vs (GST+ADM)	(DIT+ADM) vs ADM	(GST+ADM) vs ADM	(DIT+ADM) vs (GST+ADM)
	χ^2, *p*	*p*	*p*	*p*	etimate, std.error, *p*	etimate, std.error, *p*	etimate, std.error, *p*
1-month posttreatment	4.58, 0.101	0.203	1.000	0.629	1.46,1.11,0.196	0.58,0.81,0.477	0.87,1.04,0.405
3-month posttreatment	1.49, 0.475	1.000	1.000	1.000	0.7,1.07,0.514	0.03,0.71,0.963	0.67,1.05,0.526
6-month posttreatment	4.21, 0.122	0.185	1.000	0.715	5.03,629.09,0.994	0.35,0.71,0.619	4.68,629.09,0.994
12-month posttreatment	2.21, 0.330	1.000	1.000	1.000	1.25,0.94,0.194	0.18,0.83,0.829	1.06,0.91,0.254

DIT + ADM, DIT in combination with antidepressant medication group; GST + ADM, general supportive psychotherapy in combination with antidepressant medication group; ADM, antidepressant medication alone group.
